# Place de l’embrochage fasciculé selon Hackethal dans le traitement des fractures de l’humérus: à propos de 80 cas

**DOI:** 10.11604/pamj.2016.24.253.9794

**Published:** 2016-07-19

**Authors:** Omar Margad, Jalal Boukhris, Hicham Sallahi, Mohamed Daoudi, Ouahb Azriouil, Khalid Koulali

**Affiliations:** 1 Service de Traumatologie Orthopédie De l’Hôpital Militaire Avicenne, Marrakech, Maroc

**Keywords:** Fracture de l′humérus, embrochage, Hackethal, Humerus fracture, nailing, Hackethal

## Abstract

Le débat principal autour des fractures de l’humérus se centre sur les indications thérapeutiques car tout type de stabilisation orthopédique ou chirurgicale trouve des défenseurs parfois inconditionnels et véhéments. A travers cette étude rétrospective, nous avons revu 80 patients traités initialement par embrochage fasciculé centromédullaire selon Hackethal au service de traumatologie orthopédie de l’hôpital militaire Avicenne entre janvier 2000 et janvier 2012. Les fractures ont été classées selon la classification AO et selon la classification de Hackethal modifiée par De La Caffinière. L’évaluation fonctionnelle a utilisé la classification de Stewart et Hundley modifiée. Nous avons obtenu 60 très bons résultats, 6 bons résultats, 2 assez bons résultats et 2 mauvais résultats. Les deux mauvais résultats étaient des cas de pseudarthrose. Nous n’avons pas noté de paralysie radiale iatrogène, ni d’infection, ni de migration de broches. Le délai moyen de consolidation a été de 9semaines et 6 jours. C’est une méthode fiable, de réalisation facile et à faible cout économique, qui fournit une bonne stabilisation du foyer de fracture permettant ainsi la mobilisation précoce avec des résultats fonctionnels excellents.

## Abstract (en)

The main debate regarding humerus fractures focuses on their therapeutic indications because any type of orthopedic or surgical stabilization finds unconditional and sometimes vehement defenders. We conducted a retrospective study of 80 patients treated initially with Hackethal’s technique of intramedullary bundle nailing in the Department of Orthopaedics and Traumatology at Avicenne Military Hospital between January 2000 and January 2012. The fractures were classified according to AO classification and Hackethal classification (modified by De La Caffinière). Functional assessment was based on the modified Stewart and Hundley classification. We got 60 very good outcomes, 6 good outcomes, 2 quite good outcomes and 2 poor outcomes. The two cases with poor outcomes had pseudarthrosis. We noted no iatrogenic radial nerve palsy, infection or pin migration. The average time for union was 9 weeks and 6 days. This is a reliable, easy to perform and low cost technique, which provides a good stabilization of the fracture site, allowing early mobilization with excellent functional outcomes.

## Introduction

Les fractures de l’humérus ne sont pas rares, toute localisation confondue elles viennent au troisième rang des fractures du membre supérieur [1]. Leur diagnostic est facile. En revanche les modalités de leur traitement sont loin de faire l’unanimité. L’embrochage fasciculé d’Hackethal [2] fait partie des nombreux moyens d’ostéosynthèse proposés. Le but de notre étude est d’analyser les résultats de cette technique pour en apprécier les indications, les avantages et les limites.

## Méthodes

Il s’agit d’une étude rétrospective descriptive menée au service de traumatologie orthopédie de l’hôpital militaire Avicenne de Marrakech durant une période de 12 ans s’étalant entre Janvier 2000 et Janvier 2012. Ont été inclus dans cette étude 80 patients présentant des fractures humérales non pathologiques et récentes traitées initialement par embrochage centromédullaire selon Hackethal. 10 patients ont été exclus de cette étude, car ayant été perdus de vue. Les résultats ont été évalués chez 70 patients (87,5%). Des variables d’ordre épidémiologique, clinique, paraclinique, thérapeutique et évolutif ont été analysées en se basant sur une fiche d’exploitation et la convocation des malades. Il y avait 60 hommes et 10 femmes. L’âge moyen des patients était de 35 ans avec des extrêmes de 13 ans et 65 ans. La fracture a intéressé le coté droit chez 28 patients et le coté gauche chez 42 patients avec 42,8% de fractures du coté dominant. Les circonstances de survenue étaient: 50 cas d’accident de circulation (74,5%), 10 cas d’accident domestique, 6 cas d’accident de travail, 2 cas d’accident de sport, et 2 cas d’agression. Le siège de la fracture a été déterminé selon la classification d’Hackethal modifiée par De La Caffinière (Tableau 1): 2 fractures D2; 8 fractures D3; 42 fractures D4 et 8 fractures D5. Le type de trait de fracture a été précisé selon la classification de l’AO (Tableau 2): 6A1; 18A2; 34A3; 4B1; 6B2; 2C2. Les fractures les plus fréquentes étaient de type A3 en zone moyenne D4. Dans 30 cas (42,85%) il y avait une ou plusieurs lésions associées dont 8 polytraumatisés, 10 traumatismes étagés du membre supérieur et 12 fracture du membre controlatéral. La fracture était ouverte chez 4 patients: 2 stade I et 2 stade II selon la classification de Cauchoix et Duparc. Une paralysie radiale sensitivomotrice post-traumatique a été constatée chez 6 patients. Le délai moyen entre le traumatisme et la chirurgie était de 4 jours. L’anesthésie générale a été effectuée dans 68 cas et le bloc plexique dans 2 cas. 60 patients étaient installées en décubitus dorsal et 10 en décubitus latéral. L’abord se faisait par une incision postérieure débutant 2 centimètre au dessus de la fossette olécranienne, le tendon tricipital était incisé longitudinalement. La trépanation osseuse de 1 centimètre sur 2 à 3 centimètres était réalisée au dessus de la fossette. Un maximum de broches de diamètre allant de 1,8 à 3 mm était introduit dans le canal médullaire (au minimum 2 et au maximum 5). Ces broches étaient auparavant épointées et légèrement béquillées, leur passage au niveau du foyer de fracture et leur divergence dans la tête humérale se faisaient sous contrôle de l’amplificateur de brillance. Le foyer de fracture était ensuite impacté. Le foyer de fracture a été abordé dans 12 cas dont les 6 cas de paralysie radiale post-traumatique. Le montage était ensuite complété par une immobilisation type Mayo Clinic pour 21 jours. La rééducation commençait dès que l’indolence est obtenue par la mobilisation passive puis active de l’épaule, du coude, du poignet et des doigts, en excluant toutes les manœuvres douloureuses ou forcées. Les patients étaient revus tous les mois jusqu’à 6 mois post-opératoire, puis tous les trois mois. Le résultat fonctionnel était enregistré au moins 6 mois après l’opération. Nous avons utilisé la classification de Stewart et Hundley modifiée (Tableau 3). Le recul moyen était de 60 mois avec des extrêmes de 12 mois et 96 mois. Nous avons retenu le délai de 6 mois pour parler de retard de consolidation et au delà de pseudarthrose.

**Tableau 1:** Localisation des fractures selon la classification d’Hackethal modifiée par De La Caffinière

Siège du trait de fracture	
D1	fracture du col chirurgical
D2	fracture métaphysaire haute
D3	fracture de la jonction 1/3 supérieur-1/3 moyen
D4	fracture du 1/3 moyen
D5	fracture de la jonction1/3 moyen-1/3 inférieur
D6	fracture métaphysaire basse

**Tableau 2:** Classification AO des fractures de l’humérus

Type du trait de fracture	
A1	Fracture spiroïde
A2	Fracture oblique
A3	Fracture transversale
B1	Fracture spiroïde avec un 3° fragment
B2	Fracture oblique avec un 3° fragment
B3	Fracture à quatre fragments
C1	Fracture spiroïde à plusieurs fragments
C2	Fracture bifocale

**Tableau 3:** Évaluation fonctionnelle suivant la classification de Stewart et Hundley modifiée

Très bon	Absence de douleur Mobilité normale de l’épaule et du coude Bon alignement radiologique
Bon	pas de douleur ou douleur climatique Raideur de l’épaule et du coude inférieur à 20° Cal vicieux inférieur à 20°
Assez bon	douleur peu importante Raideur de l’épaule et du coude entre 20° et 40° Cal vicieux supérieur à 20°
Mauvais	Douleur persistante Raideur de l’épaule et du coude supérieure à 40° Pseudarthrose

## Résultats

Comme complications postopératoires nous avons noté 4 cas (5,7%) de trouble neuroalgodystrophique, nous n’avons pas eu de fractures iatrogènes, ni de paralysie radiale post opératoire, ni d’infection au niveau de l’orifice d’entrée des broches. Aucune migration de broches à l’épaule ou au coude n’a été relevée. Sur les 70 patients, nous avons noté 4 cas (5,7%) de retard de consolidation, il s’agissait de patients dont le foyer de fracture a été abordé, 2 cas (2,8%) de pseudarthrose aseptique, il s’agissait de fracture de type A3 en zone D5 ostéosynthèsées par 2 broches de 2,5 mm. La consolidation a été obtenue dans 68 cas (97%). Le délai moyen de consolidation a été de 9 semaines 6 jours avec des extrêmes de 7 semaines à 20 semaines. Nous avons utilisé la classification de Stewart et Hundley modifiée pour l’évaluation des résultats fonctionnels (Tableau 3), nous avons eu 60 cas (85,9%) de très bons résultats, 6 cas (8,5%) de bons résultats, 2 cas (2,8%) de résultats assez bien et 2 cas (2,8%) de mauvais résultats. Ces derniers étaient des cas de pseudarthrose. Les 6 cas de paralysie radiale post-traumatique ont récupéré, ils ont eu de bons résultats. Sur le plan fonctionnel, l’utilisation du membre supérieur atteint était possible au-dessus de la tête pour 90% des opérés. La mobilité de l’épaule était normale dans 62 cas, déficitaire de 20° en flexion et abduction en 6cas et limité dans un cas (déficitaire de 60° en flexion et abduction). La mobilité du coude était normale dans 66 cas, déficitaire de 20° d’extension dans 4 cas. L’ablation des broches a été faite avec un délai moyen de 8 mois. Au dernier recul, 64 patients (88,5%) ont repris leur activité antérieure.

## Discussion

**Figure 1:**
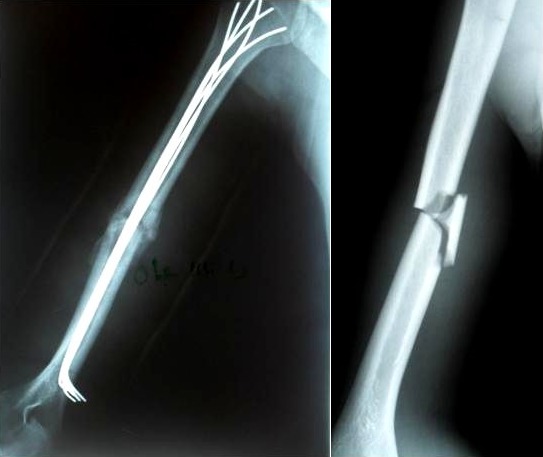
Embrochage correct: remplissage diaphysaire, divergence des broches

Ce travail a exclu les méthodes opératoires dérivées de l’embrochage fasciculé selon Hackethal mais utilisant un matériel d’ostéosynthèse différent ou dans la voie d’abord de la cavité médullaire. L’épidémiologie de notre série est sans particularités. Elle confirme que les fractures humérales atteignent les hommes jeunes lors d’un accident de la circulation et les femmes plus âgées après une chute simple. Elle montre également que les fractures medio diaphysaires sont les plus fréquentes [3]. Notre taux de 8,57% de paralysie radiale post-traumatique est comparable à la moyenne de la littérature: 8,6% pour Diémé [4], 7,73% pour Putz [5], 10% pour Coudane [6]. Comme De Mourgues [7] l’abstention en urgence et une exploration entre 3,5 et 4 mois en cas de non récupération a été notre attitude devant ces paralysies radiales. Déburge et Delisle [8] et Holstein et Lewist [9] préconisent une exploration systématique face à ces paralysies radiales. Aucune interruption du nerf radial n’a été retrouvée. La classique section du nerf radial par un fragment osseux est rare. Bezes [10] sur 17 paralysies radiales explorées n’avait noté qu’une seule rupture. Dans la littérature comme dans cette série, l’embrochage selon Hackethal se caractérise par un faible pourcentage de complications infectieuses ou neurologiques. En dehors de la série d’André [11], les autres séries rapportent des taux de pseudarthrose qui sont comparables au notre qui est de 2,8%, 2% pour Putz [5], 4,6% pour Gayet [12]. Cependant, il est de 27,6% pour André [11] et de 24% pour Gayet [13]. La principale explication est l’erreur technique en particulier le défaut d’impaction du foyer. Le grand nombre de polytraumatisés et le nombre élevé de traumatisme à haute énergie qui caractérise ce genre de collectif reste l’une des explications. Contrairement à ses séries, nous n’avons pas noté de migration de broches, elle représentait 7% pour Gayet [13], en rapport souvent avec un défaut de remplissage de la cavité médullaire, ou de blocage au niveau de la fenêtre corticale ou de divergence épiphysaire proximale. Cette complication relativement bénigne impose cependant une ablation précoce du matériel sous réserve d’une consolidation complète. Les délais de consolidation sont conformes à ceux de la littérature. 9,6 semaines pour notre série, 9,4 semaines pour Durbin [14] et 8,5 semaines pour Putz [5] ainsi que pour Freslon [13]. Nous avons rapporté 5,7% de déficit d’extension du coude, ce qui est comparable avec la littérature, 25% pour Putz [5]. Des travaux biomécaniques ont expérimentalement démontré la relative instabilité d’un embrochage huméral qui ne contrôle en fait que partiellement les contraintes rotatoires Henley [15]. L’absence de contact inter fragmentaire associé à une mobilisation précoce du membre impose au foyer de fracture des contraintes mécaniques au-delà du seuil de tolérance nécessaire au déroulement d’une ostéogenèse efficace. La simplicité technique de l’embrochage n’est qu’apparente. Les opérateurs « seniors » doivent enseigner au plus jeunes les critères incontournables du succès d’une telle technique foyer fermé. Lorsque ses règles sont respectées et que l’opérateur possède une bonne expérience personnelle, l’embrochage devient une technique fiable, rapide et sure Gayet [12] (Figure 1).

## Conclusion

Par rapport aux ostéosynthèses par clous ou par plaques, le cout de l’embrochage fasciculé selon Hackethal reste modeste et son innocuité est importante même lors de l’ablation du matériel. De plus, par rapport au traitement orthopédique, il apporte du confort au patient. Il se veut avant tout être une solution de compromis entre les méthodes orthopédiques et les ostéosynthèses rigides. C’est une technique rapide à mettre en œuvre mais qui nécessite l’expérience du foyer fermé. Le taux de pseudarthrose doit être diminué par une réalisation rigoureuse: nombre de broches maximum pour un auto blocage endo médullaire et bonne impaction du foyer de fracture.

### Etat des connaissances actuelles sur le sujet

- Les fractures de l’humérus ne sont pas rares;
- Leur traitement ne fait pas l’unanimité, de nombreux moyens d’ostéosynthèse sont proposés dont l’embrochage fasciculé selon Hackethal.

### Contribution de notre étude à la connaissance

- L’embrochage fasciculé selon Hackethal demeure une méthode efficace et fiable;
- De réalisation facile et à faible cout économique, l’embrochage fasciculé selon Hackethal permet d’obtenir des résultats fonctionnels excellents.
